# Effects of Steam Sterilization and Recycling on the Mechanical and Surface Properties of 3D-Printed Biodegradable PLA and Re-PLA Materials

**DOI:** 10.3390/polym17192590

**Published:** 2025-09-25

**Authors:** Yunus Karayer, Şakir Altınsoy, Gökçe Koç, Diyadin Can, Yunus Emre Toğar

**Affiliations:** 1 Biomedical Engineering Department, Faculty of Engineering and Architecture, Istanbul Yeni Yuzyil University, Istanbul 34010, Turkey; bmm.yunuskarayer@gmail.com (Y.K.); gokce.koc@yeniyuzyil.edu.tr (G.K.); diyadin.can@yeniyuzyil.edu.tr (D.C.); 2 Biomedical Engineering Department, Institute of Graduate Studies, Istanbul University-Cerrahpasa, Istanbul 34320, Turkey; 3 Mechatronics Program, Vocational School, Istanbul Beykent University, Istanbul 34500, Turkey; emretogar@beykent.edu.tr; 4 Mechanical Engineering Department, Faculty of Mechanical Engineering, Yıldız Technical University, Istanbul 34349, Turkey

**Keywords:** PLA, 3D printer, sterilization, recycling, mechanical properties, biocompatibility

## Abstract

Polylactic acid (PLA) is an eco-friendly polymer known for its biodegradability and biocompatibility, yet its properties are sensitive to recycling and sterilization. These processes may cause chain scission and structural irregularities, leading to reduced strength, brittleness, or unpredictable deformation. In this study, PLA and recycled PLA (Re-PLA) specimens were produced by FDM 3D printing with different infill rates (25%, 50%, 75%), layer thicknesses (0.15, 0.20, 0.25 mm), and printing orientations (0°, 45°, 90°). Steam sterilization at 121 °C and 1 bar for 15 min simulated biomedical conditions. Mechanical, surface, degradation, and biocompatibility properties were examined using three-point bending, roughness measurements, SEM, and cell viability tests. Results showed that infill rate was the main parameter affecting flexural strength and surface quality, while orientation increased roughness. Sterilization and recycling made deformation less predictable, particularly in St-Re-PLA. SEM revealed stronger bonding at higher infill, but more brittle fractures in PLA and Re-PLA, while sterilized specimens showed ductile features. No visible degradation occurred at any infill level. Regression analysis confirmed that second-order polynomial models effectively predicted flexural strength, with layer thickness being most influential. These findings provide critical insights into optimizing PLA and Re-PLA processing for biomedical applications, particularly in the production of sterilizable and recyclable implantable devices.

## 1. Introduction

Advancements in medical technologies are closely linked to the continuous pursuit of innovative solutions aimed at improving patient care. One of the primary challenges in biomedical engineering is the design of safe and durable biomaterials for use in medical devices and implants. The development of medical implants is a highly complex process that requires extensive research, precision, and meticulous execution at every stage. In recent years, additive manufacturing (AM) technologies have emerged as effective methods for implant production [1,2,3]. These techniques enable the fabrication of polymeric structures suitable for implantology while offering a high degree of design flexibility. Among the most common additive manufacturing techniques, Fused Deposition Modeling (FDM) is based on the controlled extrusion and bonding of thermoplastic materials in successive layers. Compared to conventional plastic processing technologies (e.g., extrusion, injection molding), AM enables the production of geometrically complex, fully customized products in a simple, rapid, and cost-effective manner [4]. Such personalized medical solutions present significant opportunities to improve patients’ quality of life. In particular, 3D printing allows for the cost-effective and high-precision fabrication of patient-specific anatomical structures, implants, and surgical guides [5].

Biomedical materials are subject to increasingly stringent requirements in terms of biocompatibility and immune system tolerance, as the introduction of foreign substances into the body can invariably trigger defensive responses. Such interventions may lead to inflammation, allergic reactions, and even the onset of carcinogenic processes [6]. Polymeric materials can be used either temporarily or permanently within the human body, and many single-use surgical devices (e.g., screws) are designed specifically for use during a single surgical procedure before being discarded. In this context, technological progress and advances in materials science have made the use of biodegradable polymeric materials of either natural or synthetic origin particularly attractive for the 3D printing of implants [7,8].

One of the most widely used materials in 3D printing is polylactic acid (PLA), a sustainable thermoplastic that is biocompatible, biodegradable, and FDA-approved, making it highly suitable for medical applications. PLA’s low glass transition temperature (~58 °C) and melting point (~175 °C) render it well-suited for 3D printing, and it has found widespread application in both engineering and biomedical fields [9]. Produced from renewable resources such as corn and sugarcane, PLA offers key advantages as an implant feedstock due to its non-toxicity, biocompatibility, and biodegradability [10,11]. Today, PLA is considered one of the most preferred sustainable polymers thanks to its bio-based origin, biodegradability, and recyclability [12,13]. However, the increasing adoption of 3D printing has also led to the generation of significant production waste, posing a notable environmental concern. Waste can result from the removal of support structures or from defective prints that cannot be reused, raising issues regarding environmental sustainability. According to the Plastics Europe (2022) report, approximately 20,000 tons of 3D printing waste are generated annually in Europe alone. This has necessitated the recycling and reuse of 3D printing materials, encouraging the adoption of recycled PLA (Re-PLA) [14]. Recycling waste filaments not only reduces environmental impact but also offers economic benefits. The recycling process involves collecting, shredding, remixing, homogenizing, and re-extruding waste material into filament form, thereby preventing raw material loss and reducing production costs. Many commercially available filaments suitable for 3D printing, including biocompatible polymers such as PLA and Re-PLA, are now widely accessible.

Nevertheless, for products such as implants or surgical devices that directly or indirectly come into contact with human tissue, compliance with sterilization requirements is critical [15]. Sterilization methods for effectively eliminating microorganisms in medical devices include chemical (e.g., ethylene oxide, hydrogen peroxide), mechanical (e.g., filtration), and physical (heat and radiation) techniques. Among these, heat sterilization is often preferred due to its ability to eradicate microorganisms without leaving toxic residues, its procedural simplicity, and its availability in most hospitals [16]. However, for polymeric materials, the potential effects of sterilization on mechanical and surface properties must be carefully evaluated.

One of the main challenges during sterilization is maintaining the mechanical integrity of polymeric materials. Exposure to high temperature and humidity can induce structural changes, adversely affecting their performance in medical applications. Therefore, sterilization methods and process parameters must be carefully optimized within the material’s processing window. Although PLA exhibits high strength and stiffness, it is inherently brittle. Consequently, its limited resistance to sterilization processes may restrict its widespread use in single-use surgical devices.

In this regard, Frizziero et al. reported that PLA-based 3D-printed components sterilized by moist heat in an autoclave largely retained their geometric integrity [17]. However, more pronounced deformations were observed in smaller or geometrically complex specimens. Notably, the study did not include direct mechanical testing, and assessments were based on thermal deformation and visual observations. Conversely, Zbyrad et al. demonstrated that changes in mechanical properties after steam sterilization vary depending on the type of polymer used, with certain materials such as PEEK and PET-G HT100 exhibiting high resistance to elevated temperatures [18].

While some studies have investigated only the effects of steam sterilization, others have examined the influence of manufacturing parameters (e.g., layer thickness, infill density, printing orientation) on mechanical properties without applying sterilization. For instance, Günay et al. analyzed the effects of parameters such as infill rate, printing speed, and raster angle on the tensile strength and surface quality of PLA specimens without sterilization [19]. Accordingly, Table 1 presents a comparative summary of studies that have investigated the effects of both steam sterilization and manufacturing parameters on the mechanical properties of PLA and Re-PLA.

An examination of Table 1 indicates that, in the current literature, not only the filament type but also various parameters associated with the printing process play a decisive role in determining the mechanical properties of 3D-printed components. These parameters include layer thickness, build orientation, printing speed, and infill density, with each variable either individually or in combination having a significant impact on the strength of the printed part. Although some studies have explored the influence of these parameters, analyses focusing specifically on recycled materials remain scarce. There is a clear need for research that evaluates recycled feedstocks in conjunction with manufacturing parameters. Moreover, despite the growing interest in 3D-printed products in recent years, studies investigating the structural and mechanical changes in Re-PLA-based 3D-printed parts after moist and dry heat sterilization are limited. Similarly, research that considers the combined effects of printing parameters such as layer thickness, build orientation, printing speed, and infill density together with sterilization is also limited.

Understanding the effects of sterilization on the properties of biomaterials used in implantology is critical to ensuring the safety, durability, and performance of these materials in clinical applications following sterilization. Therefore, in this study, both virgin polylactic acid (PLA) and recycled PLA (Re-PLA), which are biocompatible materials, were used to fabricate specimens via 3D printing under varying manufacturing parameters, and their mechanical performance was comprehensively analyzed before and after sterilization. In the experimental design, infill density, layer thickness, and build orientation were selected as the primary manufacturing parameters, and their effects on the flexural strength and surface quality of the specimens were examined in detail. To elucidate the microstructural origins of fracture behavior, scanning electron microscopy (SEM) analyses were performed on fracture surfaces, enabling the characterization of fracture modes at the microstructural level. The experimental results were theoretically supported through statistical analyses and mathematical modeling, allowing for a direct comparison between experimental and predicted results to validate the model’s accuracy. These modeling efforts facilitated the identification of optimal manufacturing parameters and contributed to reducing the number of required experimental trials.

Furthermore, although PLA’s biocompatibility is well established in the literature, it was hypothesized that melt extrusion-based recycling steps could affect the biocompatibility, degradation, and potential leaching behavior of Re-PLA through chain scission and the formation of low-molecular-weight species. Accordingly, biocompatibility, degradation, and leaching tests were conducted on Re-PLA. The results were comparatively analyzed alongside similar studies in the literature, and the findings from this research were interpreted to provide original contributions to the field. Overall, this study offers a comprehensive evaluation in which critical factors such as sterilization, recycling, and manufacturing parameters are jointly assessed to ensure the safe use of biodegradable and sustainable polymers in medical applications.

## 2. Materials and Methods

### 2.1. Materials and 3D Printing Process

**Figure 1:**
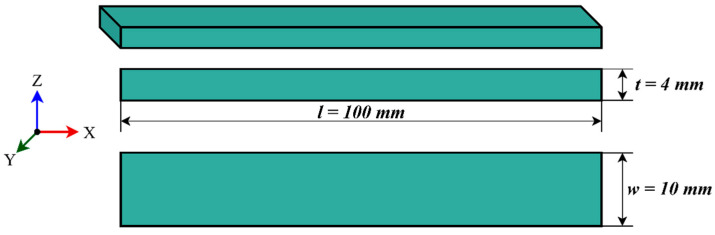
Technical drawing of the specimen.

The polylactic acid (PLA) material used in this study was the “PLA-PLUS” filament from the ELAS-3B brand. The recycled polylactic acid (Re-PLA) specimens were produced by recycling the same material. In the production of Re-PLA specimens, filaments obtained by recycling defective products produced in 3D printers with PLA PLUS filaments were used. The main properties of PLA are as follows: density 1500 kg/m^3^, elastic modulus 5.9 GPa, Poisson’s ratio 0.4, maximum tensile strength 60 MPa, and tensile elongation 2%. All specimens were manufactured using the Fused Deposition Modeling (FDM) method on a Creality CR-6 SE 3D printer (Shenzhen Creality-Chine, Shenzhen, China). Figure 1 shows the technical drawing of the test specimen used in the study. In the experimental study, infill rates (25%, 50%, and 75%), layer thicknesses (0.15 mm, 0.20 mm, and 0.25 mm), and printing orientations (0°, 45°, and 90°) were used as production parameters. In this study, a nozzle temperature of 200 °C, a bed temperature of 60 °C, a printing speed of 50 mm/s, and the cooling fan in the “on” mode were used. For each material, 27 parameter combinations were tested in triplicate (*n* = 81), yielding a total of 324 specimens across the four materials (81 × 4). Each parameter was systematically varied to examine its impact on the mechanical properties. Table 2 presents the list of materials, reagents, and their sources used in this study.

### 2.2. Recycling Steps

In this study, as part of the recycling process, defective parts generated during the production phase of 3D printers were collected and initially cleaned. Three-dimensional printing waste was cleaned by washing it under industrial conditions at 85 °C with a solution containing 1% NaOH and 3% active surface solvent. Following the cleaning process, the materials were dried under vacuum. Recycled filament production was carried out in a Rondol Microlab twin screw extruder with an L/D ratio of 20 at a rotation speed of 60 rpm.

### 2.3. Sterilization Process

Sterilization is a fundamental step used in the production process of biomaterials or medical devices that will come into contact with the human body, as well as in the reuse of medical devices, to prevent complications such as infection or implant rejection [26]. Heat sterilization is based on the destruction of the vital metabolic and structural components of microorganisms through heat and oxidative processes. The most common protocol in clinical applications is autoclaving, which involves exposing materials to pressurized steam at temperatures between 121–136 °C for specific durations [27]. However, for biopolymers and many thermoplastics (e.g., PLA, PLGA, some bio-based polymers), this process may carry risks such as hydrolysis, chain scission, and physical degradation, leading to undesirable changes in mechanical, thermal, and morphological properties. Nevertheless, the literature reports that PLA can be sterilized by autoclaving under certain conditions and does not exhibit cytotoxicity. Although increases in hydrophilicity or changes in surface roughness have been observed, no significant inflammatory response has been reported [28,29].

A key challenge in sterilization is preserving the mechanical integrity of polymeric materials. Exposure to high temperatures and humidity can cause structural changes that may adversely affect the performance of materials in medical applications. Therefore, in order to ensure both sterility and mechanical stability, the sterilization method and process parameters must be carefully optimized within the material’s processing window.

**Figure 2:**
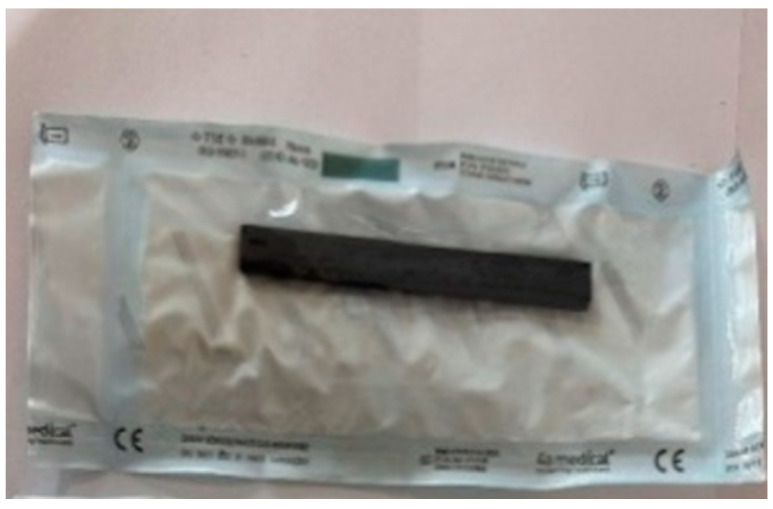
Specimen after the sterilization process.

In this study, specimens were fabricated from PLA and recycled PLA (Re-PLA) using fused deposition modeling (FDM). For each parameter combination infill rate (25%, 50%, 75%), Layer Thickness (0.15, 0.20, 0.25 mm), and printing orientation (0°, 45°, 90°) specimens were autoclaved at 121 °C and 1 bar for 15 min in medical grade sterilization pouches. The sterilization process was performed using the ERYİĞİT ERS 6613 D (2020) autoclave device. After sterilization, PLA and Re-PLA specimens are denoted as St-PLA and St-Re-PLA, respectively. Figure 2 illustrates representative sterilization packages.

### 2.4. Characterization Study

The three-point bending test is a standard mechanical testing method used to determine the flexural strength, modulus of elasticity, and fracture behavior of a material. In this test, the specimen is placed between two support points, and a single load is applied at its center, causing it to fracture or deform. In this study, the tests were conducted using a Zwick Proline three-point bending tester, according to the ISO 178:2019 standard [30]. The distance between the supports was set to 64 mm, the bending speed to 2 mm/min, and the ambient temperature was maintained at 21 ± 3 °C. Three specimens were tested for each parameter.

Surface roughness is considered an important criterion for evaluating a material’s surface quality and the effects of manufacturing process parameters on the surface. In this study, surface roughness measurements were performed using a Mitutoyo Surftest SJ-210 (Mitutoyo, Kawasaki, Japan) surface roughness device. The results were obtained using the “Surface Roughness Tester SJ-210/310/410” software. Previously tested three-point bending specimens were used in the surface roughness test. Ra (arithmetic mean deviation of the profile) values were measured to determine the surface roughness data of the test specimens. Before testing, the surfaces were thoroughly cleaned, and measurements were taken at five different points on each specimen to calculate the average Ra value. Scanning Electron Microscope (SEM) imaging was conducted with the FEI QUANTA 450 FEG ESEM device (Fei Company, Hillsboro, OR, USA), using FEG 450 model. SEM images were taken at Istanbul Arel University Polymer Technologies and Composite Application and Research Center (Arel POTKAM).

### 2.5. Degradation Tests

To study how PLA, Re-PLA, St-PLA, and St-Re-PLA behave in terms of degradation, it was prepared specimens with three different infill levels: 25%, 50%, and 75%. Each of these specimens was placed in 10 mL of phosphate-buffered saline (PBS) and kept in a shaking incubator set to 37 °C and 60 rpm, which helps mimic the conditions inside the human body. It was monitored the degradation over a period of one week. At specific time points 1, 2, 3, 4, 5, 24, 48, 72, 96, 120, 144, and 168 h we took 3 mL of solution from each container. To keep the volume stable, the removed liquid was replaced with 3 mL of fresh PBS. Each collected sample was then analyzed using a UV-Vis spectrophotometer, scanning between 200 and 800 nm, to check if any material had been degraded from the specimens over time.

### 2.6. Cytotoxic Tests

The biocompatibility testing was carried out using L929 mouse fibroblast cells with the MTT assay; this is the test where living cells convert a dye into colored crystals. The tested samples were made from PLA, recycled PLA (Re-PLA), sterilized PLA (St-PLA) and sterilized–recycled PLA (St-Re-PLA) at infill rates of 25%, 50%, and 75%. First, we sterilized all the specimens in an autoclave. After sterilization, 5 mL of serum-free DMEM F12 medium was placed over each sample and they were incubated at 37 °C for seven days. On days 1, 4, and 7, a portion of the medium was removed and stored in the fridge at 4 °C until the cytotoxicity tests were ready to run.

For the test itself, L929 cells were grown in 25 cm^2^ flasks until they covered about 80% of the surface. Then the cells were counted and the total number was calculated with the following Equation (1).

(1) $$\begin{array}{c} Total\;Cell\;Coun=Average\;Cell\;Count\times Dilution\;Factor\times 10^{4}\\ 2\times 50\times 10000=1000000\;Cell \end{array}$$

After that, 10,000 cells were seeded in 100 µL of medium into each well of a plate. The cells were left for 24 h to attach and grow. Once that time was up, the medium was removed, washed the cells with PBS, and added 100 µL of the stored sample medium from days 1, 4, or 7. Five replicates were performed for each sample to make sure the data was reliable. Then, the cells were incubated for another 24 h. Following this, 10 µL of MTT reagent (Sigma M5655) was added to each well. The plates went into the dark for 3 h so the living cells could convert the MTT into formazan crystals. After incubation, 100 µL of DMSO (Merck) was added to dissolve the crystals, waited about 30 min, and then measured absorbance at 540 nm using a plate reader. The viability of the cells was calculated according to the equation given in Equation (2).

(2) $$\%\;Viability=\frac{Experimental\;Group\;Absorbance}{Control\;Group\;Absorbance}\times 100$$

### 2.7. Mathematical Modeling

The advancement of additive manufacturing technologies, particularly with biodegradable thermoplastics such as PLA and derivatives, has necessitated the use of mathematical modeling to rigorously analyze the effects of process parameters on mechanical and surface properties. Parameters such as infill density, layer thickness, and printing orientations exert complex, interdependent influences on material performance, and their evaluation requires robust statistical and mathematical formulations. In this context, correlation analysis and variance-based statistical methods such as ANOVA provide a foundation for identifying significant relationships among variables, while regression analysis enables the development of predictive models that capture both linear and nonlinear dependencies.

The correlation coefficient (r) is employed as a measure of the strength and direction of relationships between parameters, with values approaching +1 or −1 indicating strong positive or negative correlations, respectively, while values near zero suggest the absence of a meaningful statistical relationship. However, correlation alone cannot establish causality; thus, regression modeling becomes central in quantifying the extent to which independent variables contribute to variations in output responses such as bending strength (σ_max_) or surface roughness (Ra).

The regression framework adopted in this study extends beyond simple linear relationships by incorporating higher-order and interaction terms, yielding a generalized polynomial form (Equation (3)):

(3) $$Y=\beta_{0}+\beta_{1}X_{1}+\beta_{2}X_{2}+\beta_{3}X_{3}+\beta_{4}X_{1}^{2}+\beta_{5}X_{2}^{2}+\beta_{6}X_{3}^{2}+\beta_{7}X_{1}X_{2}+\beta_{8}X_{1}X_{3}+\beta_{9}X_{2}X_{3}$$

where Y represents the response variable of interest and $X_{i}$ denote the process parameters under investigation. The estimation of regression coefficients was performed using Wolfram Mathematica, which provides both parameter values and statistical significance tests. The predictive capacity of the models was subsequently evaluated through established statistical metrics, including the coefficient of determination (R2), mean absolute error (MAE), and root mean square error (RMSE). Collectively, these measures provide a comprehensive assessment of the model’s explanatory power, its average predictive deviation, and its sensitivity to larger discrepancies.

The application of this modeling framework to experimental datasets derived from PLA and its derivatives demonstrated that the interaction between material type and processing parameters is highly significant in determining deformation behavior. Regression-based models not only allowed for the quantification of individual parameter effects but also facilitated a broader understanding of interdependencies within the manufacturing process. Moreover, by averaging the regression coefficients obtained across different material systems, a composite predictive model was constructed. This pooled model representation was found to be effective in capturing general trends across diverse material classes, thereby offering a more holistic approach for the optimization of additive manufacturing processes.

## 3. Results and Discussion

In this section, the results of the bending test, deformation, surface roughness, SEM images, degradation test, cytotoxicity test, and mathematical modeling are presented in tables and graphs. These results are then discussed and compared with recent studies in the literature. In addition, statistical and parametric analyses are carried out to show how the printing parameters affect the mechanical and surface properties of the specimens. Finally, the most suitable printing conditions that improve mechanical strength and surface quality are identified. The experimental results of maximum flexural strength, maximum deformation, and surface roughness for PLA, Re-PLA, St-PLA, and St-Re-PLA specimens are given in Table 3.

### 3.1. Three-Point Bending Test Results

**Figure 3:**
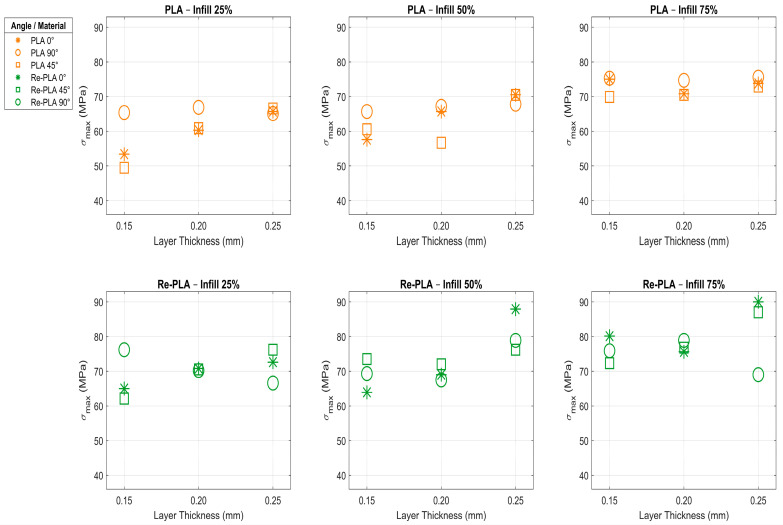
Effects of the same production parameters on the flexural strength of PLA and Re-PLA specimens.

**Figure 4:**
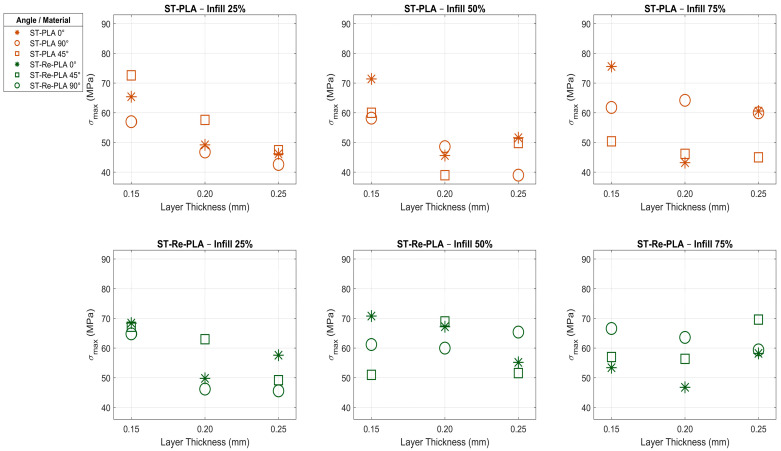
Effects of the same production parameters on the flexural strength of St-PLA and St-Re-PLA specimens.

The three-point bending test is used to evaluate the flexural strength and elastic modulus of materials. Flexural strength indicates how well a material can resist breaking under applied force, while the elastic modulus reflects the material’s stiffness and its ability to resist deformation when subjected to stress. Both parameters are critically important for assessing the strength of materials exposed to high mechanical loads, such as those used in implant applications. The flexural strength test results for PLA, Re-PLA, St-PLA, and St-Re-PLA specimens, based on the experiments and measurements performed in this study, are presented in Table 3, as given above. Figure 3 and Figure 4 show graphs created by averaging the relevant parameters, making it easier to interpret the results in Table 3.

Figure 3 shows how the same production parameters affect the flexural strength of PLA and Re-PLA specimens, while Figure 4 shows their effect on St-PLA and St-Re-PLA specimens.

When Table 3 and Figure 3 are examined, it is observed that flexural strength increases as the infill rate, printing orientation, and layer thickness of the PLA specimens increase. Increasing the infill rate from 25% to 50% led to a 5.146% increase in flexural strength, and increasing it from 50% to 75% resulted in a 13.086% increase. Increasing the layer thickness from 0.15 mm to 0.20 mm produced a 3.721% increase in flexural strength, and increasing it from 0.20 mm to 0.25 mm yielded a 5.862% increase. When evaluated according to printing orientation, increasing the angle from 0° to 45° resulted in a 2.48% decrease, while increasing it from 45° to 90° resulted in a 7.888% increase. The most influential parameter in PLA specimens was determined to be the infill rate. Because it directly alters the physical structure and internal strength of the material, it has been observed to have a more pronounced and powerful effect on flexural strength compared to other parameters [21,31,32]. The highest flexural strength was measured in a specimen with a 75% infill rate, a layer thickness of 0.25 mm, and a 90° printing orientation. In PLA, it is seen that the flexural strength values are almost the same in all printing orientations (0°, 45°, and 90°) at 75% infill rate and 2.0 mm layer thickness (Table 3, Figure 3). This can be explained by the minimisation of the internal porosity of the material at high infill rate and the formation of a more homogeneous structure. Thus, the differences in the printing orientation due to interlayer adhesion become insignificant, and the load transfer is more uniform regardless of the orientation. Similar results have been reported in the literature; it is emphasised that the effect of printing orientation on flexural strength is significantly reduced at high infill rates and the main determining parameter is the idolation ratio [33,34,35].

When the Re-PLA results presented in Table 3 and Figure 3 were examined, it was observed that flexural strength increased with higher infill rates and greater layer thicknesses, but decreased with increasing printing orientation (Table 3, Figure 3). Increasing the infill rate from 25% to 50% led to a 4.443% increase in flexural strength, and increasing it from 50% to 75% resulted in a 7.201% increase. Increasing the layer thickness from 0.15 mm to 0.20 mm yielded an approximate 2% increase in flexural strength, while increasing it from 0.20 mm to 0.25 mm led to an 8.153% increase. Regarding printing orientation, increasing the angle from 0° to 45° caused an approximate 1% decrease, and increasing it from 45° to 90° resulted in an approximate 2% decrease. It was determined that infill rate and layer thickness have a comparable impact on the flexural strength of Re-PLA specimens. Increasing the printing orientation is thought to negatively affect interlayer adhesion and the direction of load transmission, leading to a reduction in flexural strength [21,31,32]. The highest flexural strength was measured in the specimen with a 75% infill rate, a 0.25 mm layer thickness, and a 0° printing orientation.

The main reason why a similar increasing trend is observed in Re-PLA specimens at all infill rates (25, 50 and 75%) in Figure 3 is that as the infill rate increases, the internal porosity decreases, the adhesion between the layers strengthens and the load transfer becomes more homogeneous. Although chain shortening and crystallinity changes due to the recycling process limit the overall strength of the material, increasing infill rate and layer thickness compensate these negative effects and increase the flexural strength. However, since the increase in printing orientation makes load transfer difficult at the interface, it negatively affects the strength in Re-PLA [36,37].

In the St-PLA specimens, the infill rate, layer thickness, and printing orientation were observed to be effective parameters (Table 3, Figure 4). Increasing the infill rate from 25% to 50% resulted in a 4.455% decrease in flexural strength; however, increasing the infill rate from 50% to 75% resulted in a 9.456% increase. Increasing the layer thickness from 0.15 mm to 0.20 mm resulted in a ≈23% decrease in flexural strength, while increasing it from 0.20 mm to 0.25 mm resulted in a ≈0.4% increase. This suggests that a thickness of 0.20 mm may be a critical threshold, and interlayer adhesion is minimal at this value. Regarding the printing orientation, increasing the printing orientation from 0° to 45° resulted in an ≈8% decrease in flexural strength, while increasing it from 45° to 90° resulted in a ≈2% increase. In St-PLA specimens, layer thickness has been determined to have a greater effect on flexural strength [21,31,32,38]. The highest flexural strength was measured in the specimen with a 75% infill rate, a 0.15 mm layer thickness, and a 0° printing orientation.

In the St-Re-PLA specimens, it was observed that infill rate, layer thickness, and printing orientation were the key parameters influencing flexural strength (Table 3, Figure 4). Increasing the infill rate from 25% to 50% led to a 7.737% increase in flexural strength; however, increasing it from 50% to 75% caused a 3.7% decrease. Increasing the layer thickness from 0.15 mm to 0.20 mm resulted in a 6.852% decrease in flexural strength, while increasing it from 0.20 mm to 0.25 mm led to a further approximate 2% decrease. When evaluated by printing orientation, increasing the angle from 0° to 45° produced an approximate 1% increase in flexural strength, while increasing it from 45° to 90° had virtually no effect. The highest flexural strength was recorded in the specimen with a 50% infill rate, 0.15 mm layer thickness, and 0° printing orientation. Overall, no consistent trend was observed in the figures. This irregularity is thought to be the result of the specimens undergoing both sterilization and recycling processes [21,32,39].

The irregular trends seen in the St-PLA and St-Re-PLA specimens in Figure 4 are due to the fact that the sterilisation and recycling processes disrupt the polymer chain structure, weakening interlayer bonding, increasing internal stresses, and making the microstructure heterogeneous. For this reason, even if the infill rate increased, the strength did not always increase, and in some cases, on the contrary, decreases were observed. In addition, critical threshold values occurred at certain layer thicknesses, minimising interface adhesion and causing fluctuations in mechanical performance; it has been reported in the literature that such processes can cause unpredictable strength losses in biopolymers [37].

A general evaluation of the flexural strength tests performed on PLA, Re-PLA, St-PLA, and St-Re-PLA specimens reveals that production parameters such as infill rate, layer thickness, and printing orientation have varying effects on each sample type. In general, increasing the infill rate increases flexural strength in PLA and Re-PLA specimens, while increasing the printing orientation has a limited effect on PLA specimens and decreases strength in Re-PLA specimens. Increasing layer thickness generally increases flexural strength in both material types, but this increase was limited in some cases.

In St-PLA specimens, increasing the infill rate initially reduces flexural strength, followed by an increase, revealing the complex interaction of this parameter with the material’s microstructure. Furthermore, St-PLA specimens, which exhibit greater sensitivity to layer thickness, were determined to have a critical thickness of 0.20 mm. In St-Re-PLA specimens, both the infill rate and layer thickness caused a decrease in flexural strength within certain ranges. This was attributed to the negative effects of sterilization and recycling processes on material properties.

Increasing the printing orientation generally led to a slight decrease in flexural strength across all specimen types, although this effect was negligible in some cases. Based on these findings, it was determined that the most influential parameter in PLA-based specimens is the infill rate, whereas sterilization and recycling processes introduced greater variability in material behavior. Therefore, it was concluded that the production parameters for recycled and sterilized biopolymers must be optimized with greater precision and care [21,23,31,32,38].

### 3.2. Deformation Test Results

Another important piece of information obtained from the three-point bending tests is the deformation behavior of the materials. Deformation tests are fundamental engineering analyses used to evaluate the mechanical performance of a material under external loading. The elastic, plastic, and fracture behaviors of a material are quantitatively assessed through strain and stress parameters, providing critical insights into properties such as service life, toughness, and suitability for engineering applications.

**Figure 5:**
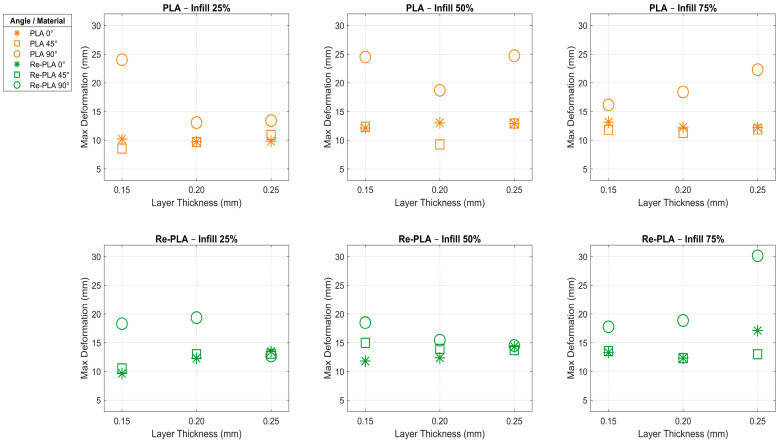
Effects of production parameters on deformation of PLA and Re-PLA specimens.

**Figure 6:**
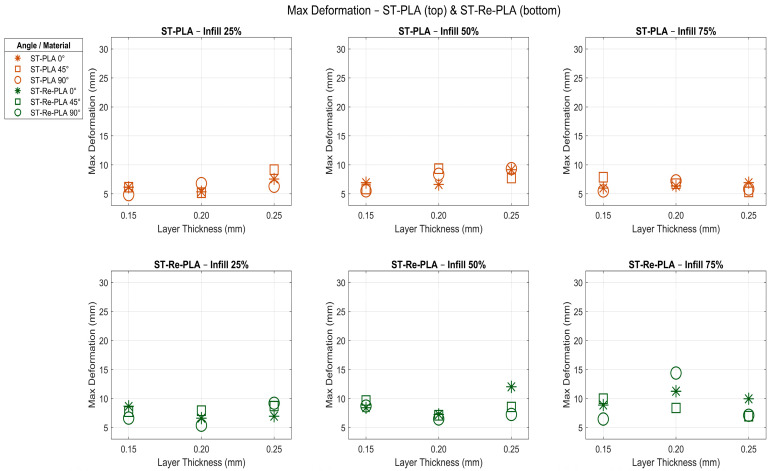
Effects of production parameters on deformation of St-PLA and St-Re-PLA specimens.

In recent years, the significance of deformation testing has grown, particularly in evaluating the performance of recycled and/or sterilized polymer materials produced using 3D printers. The deformation test results of PLA, Re-PLA, St-PLA and St-Re-PLA specimens are given in Table 3. The effects of the production parameters on the deformation of PLA and Re-PLA specimens are shown in Figure 5, while Figure 6 shows these effects for St-PLA and St-Re-PLA specimens.

According to the deformation (ε) data presented in Table 3, Figure 5 and Figure 6, the effects of the production parameters on the deformation of PLA, Re-PLA, St-PLA, and St-Re-PLA specimens were systematically evaluated. It was found that the infill rate, layer thickness, and printing orientation, which are considered production parameters, affect the deformation behavior at different rates and directions depending on the specimen type.

In PLA specimens, the deformation increased by 28.46% when the infill rate was increased from 25% to 50%, while a decrease of 7.89% was observed when the infill rate was increased from 50% to 75%. This can be attributed to the reduction in voids in the microstructure and the increase in the deformation capacity of the material under load. However, at a 75% infill rate, a decrease in deformation capacity may have occurred as the structure became more rigid. Similarly, in Re-PLA, the increase was limited to 5.88% from 25% to 50% and 14.53% from 50% to 75%. This shows that Re-PLA is not as sensitive to infill rate as PLA due to irregularities in its internal structure. There are two important factors behind the deformation differences between PLA and Re-PLA materials. The first is how the production parameters shape the microstructural voids. When the infill rate in PLA increases up to a certain level, the voids between the layers are reduced, thereby increasing the deformation capacity of the material. However, when the infill rate reaches very high levels, the structure becomes overly rigid, and this becomes a factor limiting the deformation capacity. The second is the effect of recycling and sterilisation processes on polymer chains. In Re-PLA production, as recycled polymer chains undergo thermal and mechanical processes, breaks and irregularities occur in the chain structure. This leads to weakening of interlayer bonds, an increase in internal stresses, and heterogenisation of the microstructure. As a result, an increase in parameters such as infill rate or layer thickness does not always increase the mechanical strength of the material; in some cases, decreases in strength can be observed [23,32,38].

In the sterilized specimens (St-PLA and St-Re-PLA), the deformation increase was found to proceed more irregularly with the infill rate. In St-PLA, the deformation increased by 19.61% from 25% to 50%, while it decreased by 15.31% from 50% to 75%. This suggests that the microcracks formed in the internal structure after sterilization lose their effect with filling [39,40,41,42]. In St-Re-PLA, it was observed that the increase in deformation was irregular as the infill rate increased. When the deformation behaviours of St-PLA and St-Re-PLA specimens are examined, it is seen that the values do not increase steadily as the infill rate increases, and in some cases decrease. The main reason for this situation is that sterilisation and recycling processes cause breaks in polymer chains, weakening of interlayer bonds, and irregularities in the microstructure. While the structure is normally expected to become more durable when the infill rate increases, this effect is suppressed due to the microcracks caused by sterilisation and the heterogeneous structure caused by recycling, and in some cases, even the deformation capacity decreases.

When the deformation (ε) data presented in Table 3, Figure 5 and Figure 6 are evaluated based on layer thickness, a clear trend is observed in PLA specimens. As the layer thickness increases from 0.15 mm to 0.20 mm, deformation increases by 9.45%, and by an additional 11.84% as it increases from 0.20 mm to 0.25 mm.

This behavior indicates that thinner layers tend to make the structure more brittle, whereas thicker layers enhance the material’s capacity to deform, even though the interlayer adhesion may become weaker due to reduced bonding between layers. In contrast, Re-PLA specimens exhibited much smaller changes, suggesting that they maintain a more stable structural response to changes in layer thickness. This could be related to altered molecular structure or stress relaxation effects introduced during the recycling process. For St-PLA and St-Re-PLA specimens, the deformation did not follow a regular pattern with increasing layer thickness. Particularly in St-PLA, deformation increased by 13.10% and 9.45% across the same thickness intervals, but these increases were inconsistent. This fluctuation is likely due to the weakening of interlayer bonding caused by the sterilization process, which may have degraded the surface properties of the layers or introduced microdefects. On the other hand, St-Re-PLA specimens showed a more consistent and limited change, indicating that the combined effects of sterilization and recycling resulted in a structure less sensitive to layer thickness variations.

When the same tables and figures are evaluated in terms of printing orientation, a 6.48% decrease in deformation was observed in PLA specimens as the printing orientation increased from 0° to 45°, whereas a substantial increase of 77.80% was recorded when the orientation increased from 45° to 90° (Table 3, Figure 5 and Figure 6). This significant rise is attributed to the fact that at 90° printing orientation, the applied load is perpendicular to the interlayer bonds, making deformation easier due to weaker interfacial strength. In Re-PLA specimens, a similar trend was observed, with deformation increasing by 40.34% as the printing orientation changed, although the magnitude of change was lower than in PLA. This suggests that the effect of printing orientation on deformation is more limited in recycled materials, possibly due to altered microstructure or more uniform energy dissipation [21,23,31,32,38]. For St-PLA and St-Re-PLA specimens, deformation behavior showed fluctuating patterns depending on the printing orientation. Specifically, in St-PLA, deformation increased by 4.66% between 0° and 45°, but then decreased by 6.50% between 45° and 90°. In St-Re-PLA specimens, however, the variation in deformation was minimal, indicating that the influence of printing orientation diminished following the sterilization process [32,37,38,43,44,45].

### 3.3. Surface Roughness Results

**Figure 7:**
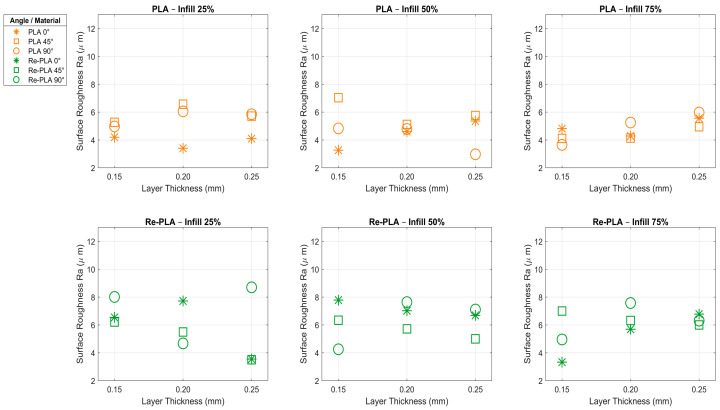
Effects of production parameters on surface roughness of PLA and Re-PLA specimens.

**Figure 8:**
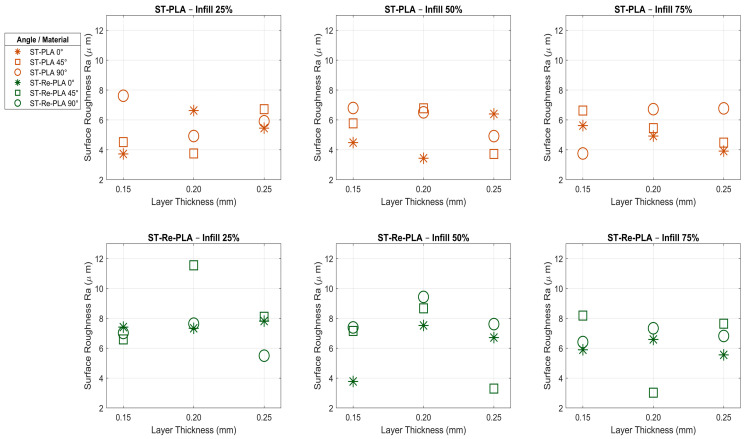
Effects of production parameters on surface roughness of St-PLA and St-Re-PLA specimens.

Surface roughness is a basic parameter that has a direct impact on the quality level, functionality, and cost-efficiency of a product. Advances in manufacturing technologies and increased research and development activities allow the optimisation of production parameters in order to achieve higher surface quality. In this context, the investigation of the surface roughness of PLA- and Re-PLA-based materials manufactured by 3D printing technology provides important information about the surface characteristics of these materials. In this study, surface roughness measurements were performed on four different specimen types, namely PLA, St-PLA, Re-PLA, and St-Re-PLA, at five different points for each specimen, and the averages of the obtained values are presented. The surface roughness test results are summarized in Table 3. At the same time, Figure 7 and Figure 8 illustrate the effects of production parameters on the surface roughness of PLA and Re-PLA specimens, and St-PLA and St-Re-PLA specimens, respectively.

When Table 3 and Figure 7 are analyzed, it is observed that the surface roughness decreases as the infill rate increases in PLA specimens, whereas it increases with increasing layer thickness. The surface roughness (Ra value) decreased by 5.143% when the infill rate was increased from 25% to 50%, and by 2.3% when it was increased from 50% to 75%. This reduction is attributed to the fact that higher infill rates minimize internal voids, resulting in a more uniform and smoother surface texture. On the other hand, increasing the layer thickness from 0.15 mm to 0.20 mm led to a 4.926% rise in the Ra value, while a further increase from 0.20 mm to 0.25 mm caused an approximate increase of 4.5%. This can be explained by the fact that thicker layers lead to more pronounced transitions between layers, which adversely affect surface quality. In terms of printing orientation, surface roughness increased by approximately 23% when the orientation was changed from 0° to 45°, but decreased by about 9% when the orientation was further changed from 45° to 90°. This behavior is likely due to the way layer deposition aligns relative to the printing orientation, which directly influences the external surface profile. As a result, it was determined that the most effective parameter on the surface roughness of PLA specimens was the infill rate, while the most negative parameter affecting the surface quality was the printing orientation [31,32]. The lowest surface roughness was obtained in the specimen with 50% infill rate, 0.25 mm layer thickness, and 90° printing orientation, while the highest Ra value was obtained in the specimen with 50% infill rate, 0.15 mm layer thickness, and 45° printing orientation.

When the data presented in the same tables and figures were analyzed according to Re-PLA specimens, it was found that there was an initial increase in surface roughness and then a decreasing trend as the infill rate and layer thickness increased (Table 3, Figure 7). The surface roughness (Ra value) increased by 2.37% when the infill rate was increased from 25% to 50% and decreased by 6.22% when the infill rate was increased from 50% to 75%. This can be explained by the fact that the surface becomes rougher due to the irregularity of the internal structure at medium filling level, while at high filling level, the surface becomes smoother due to the reduction in voids [21,23]. Increasing the layer thickness from 0.15 mm to 0.20 mm resulted in an increase of 6.278% in the Ra value, while increasing the layer thickness from 0.20 mm to 0.25 mm resulted in a decrease of approximately 4.161%. This can be attributed to the fact that after a certain thickness, layer transitions become more harmonious and reduce surface roughness [31]. In terms of printing orientation, the surface roughness decreased by ≈3% by increasing the printing orientation from 0° to 45° and increased by ≈11% by increasing the printing orientation from 45° to 90°. This increase can be explained by the fact that as the production angle reaches 90°, the layers accumulate in the vertical direction, causing more distinct surface marks [31]. The lowest surface roughness was obtained in the specimen with 75% infill rate, 0.15 mm layer thickness, and 0° printing orientation, while the highest Ra value was measured in the specimen with 25% infill rate, 0.25 mm layer thickness, and 90° printing orientation. As a result, it has been determined that the most negative parameter affecting the surface roughness of Re-PLA specimens is the printing orientation, while the infill rate and layer thickness can affect the surface quality both positively and negatively [23,31].

When the graphs and data presented in the table and figures are examined according to St-PLA specimens, it was found that the surface roughness decreased as the infill rate increased in the sterilised PLA (St-PLA) specimens, whereas the surface roughness first increased and then decreased as the layer thickness increased (Table 3, Figure 8). In addition, it has been observed that the surface roughness increases significantly with increasing printing orientation and adversely affects the surface quality [31,32]. The surface roughness (Ra value) decreased by ≈1% when the infill rate was increased from 25% to 50% and by ≈1.2% when the infill rate was increased from 50% to 75%. This can be explained by the fact that the increased infill rate makes the internal structure tighter and reduces the voids and distortions on the surface [32]. Increasing the layer thickness from 0.15 mm to 0.20 mm caused a slight increase of 0.44% in the Ra value, while increasing the layer thickness from 0.20 mm to 0.25 mm resulted in a decrease of approximately 1.98%. This may be related to the fact that after a certain thickness, the surface becomes smoother as the alignment between the layers becomes more uniform [31].

When evaluated in terms of printing orientation, the surface roughness increased by 7.3% when the angle was increased from 0° to 45°, while this increase reached 12.757% when the angle was increased from 45° to 90°. This increase is explained by the fact that as the printing orientation increases, the transition lines between the layers become more distinct and the printing direction forms a steeper angle with the surface [31]. The lowest surface roughness was obtained in the specimen with 50% infill rate, 0.2 mm layer thickness, and 0° printing orientation, while the highest Ra value was measured in the specimen with 50% infill rate, 0.15 mm layer thickness, and 90° printing orientation. As a result, it has been determined that the parameter that most affects the surface roughness of St-PLA specimens is the printing orientation, while the infill rate contributes positively to the improvement of the surface quality [31,32].

When the results obtained are analyzed according to St-Re-PLA specimens, it is seen that the surface roughness decreases as the infill rate increases (Table 3, Figure 8). On the other hand, similar to Re-PLA and St-PLA specimens, the surface roughness increases as the layer thickness increases and decreases after a certain value. The surface roughness (Ra value) decreased by 10.735% when the infill rate was increased from 25% to 50% and by ≈7% when the infill rate was increased from 50% to 75%. Increasing the layer thickness from 0.15 mm to 0.20 mm increased the Ra value by 15.478% and increasing the layer thickness from 0.20 mm to 0.25 mm decreased the Ra value by ≈14.5%. In terms of printing orientation, it was found that it only increased similarly to St-PLA specimens, the surface roughness increased by 9.55% when increasing from 0° to 45°, and increased by 1.48% when increasing from 45° to 90°. The lowest surface roughness was obtained in the specimen with 75% infill rate, 0.2 mm layer thickness, and 45° printing orientation, while the highest Ra value was measured in the specimen with 25% infill rate, 0.2 mm layer thickness, and 45° printing orientation. As a result, it has been determined that the most effective parameter on the surface roughness of PLA specimens is the infill rate, while the most negative parameter affecting the surface quality is the printing orientation [23,32].

According to the general evaluation of the surface roughness (Ra) tests shown in the graphs given in Figure 7 and Figure 8, increasing the infill rate had a decreasing effect on the surface roughness in all specimen types. The reason for this is that as the infill rate increases, the voids in the structure decrease, the material density increases, and the surface becomes smoother. Especially in St-Re-PLA specimens, this effect was much more pronounced, and a decrease of up to 10% in the roughness value was observed at high infill rates. In Re-PLA specimens, a short-term increase was observed at medium infill rates due to the more irregular internal structure, but at high infills, this situation was replaced by smooth surface formation [21,23,32].

The layer thickness parameter showed different effects depending on the specimen type. As the layer thickness increased, the surface roughness generally increased first and then started to decrease after a certain value. This can be explained as the smoothing of the surface at low thicknesses due to more precise and smoother transitions between layers, the increase in roughness at medium thicknesses as the transitions become more pronounced, and the decrease in surface fluctuations at high thicknesses as the layer alignment becomes harmonious again. Especially in St-Re-PLA specimens, an increase of 15.5% by increasing the layer thickness from 0.15 mm to 0.20 mm and a decrease of 14.5% by increasing the layer thickness from 0.20 mm to 0.25 mm provided a clear example of this trend [31].

The printing orientation was the parameter that had the most negative effect on the surface roughness in all specimen types examined. With the increase in the printing orientation, the layers are placed on the surface at a steeper angle, and this causes the layer traces to become more prominent on the surface. This increases the surface roughness and negatively affects the surface quality. Especially in PLA and St-PLA specimens, an increase of up to 23% in roughness was observed from 0° to 45°. In Re-PLA specimens, the surface roughness decreased when the printing orientation was increased from 0° to 45° and increased again when the printing orientation was increased from 45° to 90°. This indicates that the recycled material structure has a different and more complex response to the printing orientation [23,31].

When the results shown in the figures are evaluated in general, it is seen that the internal voids in the specimens decrease with the increase in the infill rate; as a result, a more homogeneous structure is obtained, and the surface quality improves. The layer thickness parameter shows a more complex effect: at low thicknesses, the more regular layer transitions contribute to the smoothing of the surface, while at medium thicknesses, the surface roughness increases due to the prominence of the transitions. However, at higher thicknesses, the roughness decreases again as the layer alignment becomes more harmonised. The printing orientation was the parameter that affected the surface quality most negatively in all specimen types. As the printing orientation increased, the layer traces became more prominent on the surface and increased the roughness. Especially in the specimens containing recycled material (Re-PLA and St-Re-PLA), more complex responses to the printing orientation were observed due to the irregular internal structure of the material.

### 3.4. SEM Analysis

**Figure 9:**
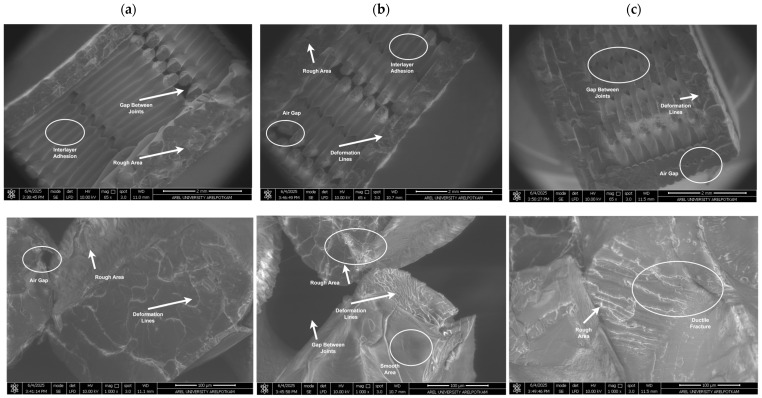
SEM images of PLA specimens with (**a**) 25%, (**b**) 50%, and (**c**) 75% infill rate.

**Figure 10:**
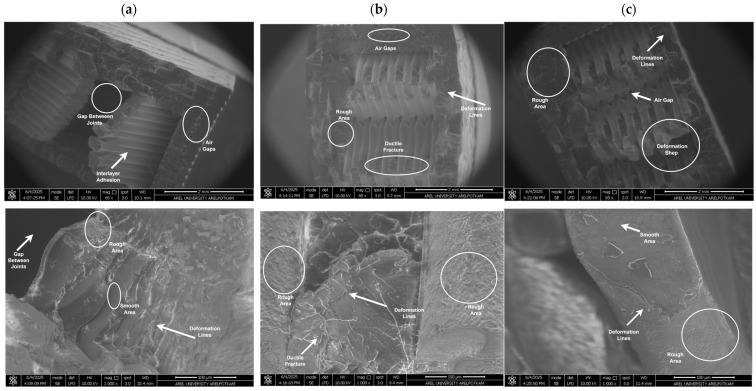
SEM images of Re-PLA specimens with (**a**) 25%, (**b**) 50%, and (**c**) 75% infill rate.

**Figure 11:**
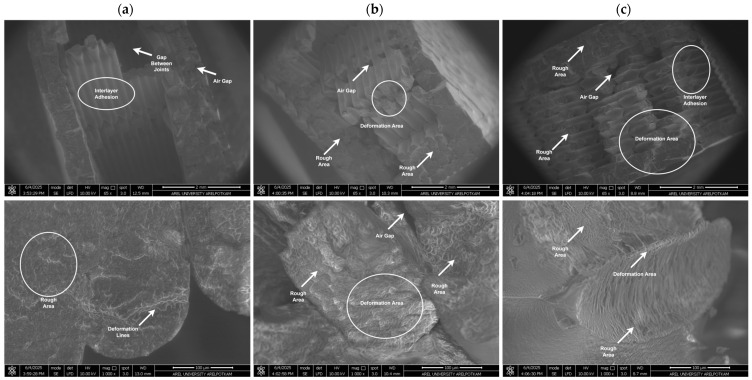
SEM images of St-PLA specimens with (**a**) 25%, (**b**) 50%, and (**c**) 75% infill rate.

**Figure 12:**
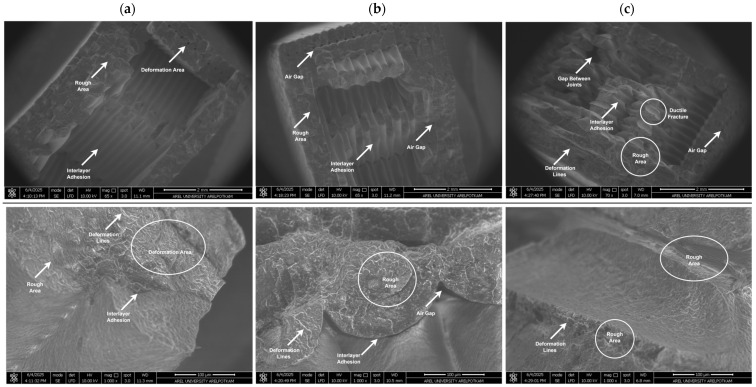
SEM images of St-Re-PLA specimens with (**a**) 25%, (**b**) 50%, and (**c**) 75% infill rate.

The analysis of fracture surfaces resulting from mechanical tests is crucial for evaluating the material’s microstructural properties and damage mechanisms. In this study, the fracture surfaces of PLA, Re-PLA, St-PLA, and St-Re-PLA specimens produced with 25%, 50%, and 75% infill rates were examined under fixed production parameters (0.2 mm layer thickness and 45° printing orientation) and analyzed using SEM imaging. Figure 9, Figure 10, Figure 11 and Figure 12 present the SEM images of fracture surfaces obtained after bending tests on PLA, Re-PLA, St-PLA, and St-Re-PLA specimens, corresponding to the three infill rates.

When the SEM images between Figure 9 and Figure 12 are examined, it is seen that the microstructural voids between the filaments (interlayer voids and trapped air gaps) are significantly reduced with the increase in the infill rate in the specimens. This is thought to be due to the more effective filling of the gaps between the extruded tracks due to the turbulent flow that occurs during the exit of the material from the nozzle [46].

The reduction in interlayer voids and air gaps has a significant impact on the mechanical performance of the material. It was observed that the fracture of PLA specimens has sharper edges and exhibits a typical brittle fracture behavior. It is also noteworthy that the inter-filament adhesion in these specimens is smooth and flawless. Similarly, in Re-PLA specimens, it was observed that the fracture was brittle and the air bubbles decreased with the increase in the infill rate and the inter-filament adhesion improved in parallel.

In St-PLA and St-Re-PLA specimens, clear signs of plastic deformation were observed on the fracture surfaces, indicating a ductile fracture behavior. Moreover, in these specimens, the filaments appeared to flatten from their original circular shape, and ductile fracture features were visible along the interlayer boundaries. This morphology suggests a reduction in surface quality. The surface roughness results were found to align well with the SEM observations. These findings are also consistent with previous studies in the literature [46,47,48].

### 3.5. Degradation Tests Results

The degradation behavior of PLA, Re-PLA, St-PLA, and St-Re-PLA specimens was checked in PBS over a full week (168 h). Since these materials are meant to be used in living systems, PBS was used as it closely resembles the body’s environment. The specimens with different infill rates (25%, 50%, and 75%) were examined using a UV-Vis spectrophotometer between 200 and 800 nm at various time points, from 1 h up to 7 days. Surprisingly, no detectable degradation was observed throughout the experiment. This likely happened because PLA is hydrophobic, so it does not dissolve in the PBS solution. This is expected, since PLA is widely used for implants due to its stability and resistance to breaking down in aqueous environments. Overall, none of the specimens released any detectable substances, regardless of the infill rate, whether the PLA was recycled or not, or whether sterilization was applied [49].

### 3.6. Cytotoxic Tests Results

**Figure 13:**
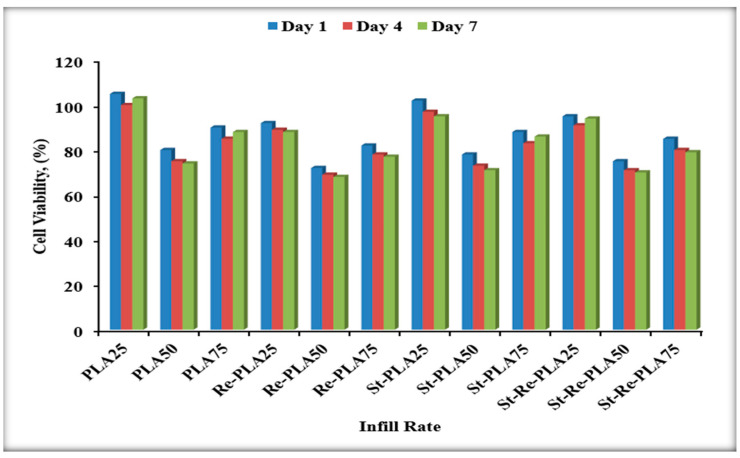
Comparison of MTT test results.

According to the measurement results of the obtained biocompatibility experiments, % viability values MTT test results are given in Figure 13 as a columnar graph. Viability percentages were determined according to the control group using the % cell viability formula. The cell viability of the control group was accepted as 100%.

The cell viability results from the MTT assay are shown in Figure 13. The control group was used as our baseline, setting their viability at 100%. Looking at the results, PLA specimens with 25% infill showed the highest values, reaching 105% on day 1, decreasing to 100% on day 4, and increasing again to 103% on day 7. Similarly, PLA at 75% infill also showed a recovery on the 7th day (90% → 85% → 88%). For PLA with 50% infill, values stayed lower (80% → 75% → 74%) without recovery.

For St-PLA, most groups decreased gradually, but at 75% infill, viability recovered from 88% on day 1, to 83% on day 4, and back up to 86% on day 7. At lower infills (25% and 50%), values continued to decline (e.g., 25% infill: 102% → 97% → 95%). For St-Re-PLA, only the 25% infill condition showed a small recovery (95% → 91% → 94%), while 50% and 75% infills continued to decline (e.g., 50% infill: 75% → 71% → 70%). For Re-PLA, all conditions showed a gradual decline with no significant recovery. For example, at 25% infill, viability decreased from 92% on day 1, to 89% on day 4, and 88% on day 7.

Overall, the ranking of materials in terms of biocompatibility was: 1. PLA > 2. St-PLA > 3. St-Re-PLA>4.Re-PLA. And the ranking of infill rates was: 25% > 75% > 50%. According to the ISO 10993-5 standard [50], if cell survival is above 70%, the material is not considered cytotoxic [50,51,52]. Based on this, all tested materials and conditions in this study can be considered non-cytotoxic and biocompatible, even though certain groups showed a decline while others exhibited recovery at the 7th day.

The different trends of the cytotoxicity tests are related to the structural changes resulting from the recycling and sterilization processes of the materials. In PLA and some St-PLA groups, cell viability tended to recover on day 7, whereas in Re-PLA, a continuous decrease was observed due to chain shortening and structural deterioration. In St-Re-PLA, recovery was limited due to the negative effects of both processes. These differences are due to the porosity, surface properties, and the level of interaction with the biological environment, and all specimens are considered non-cytotoxic and biocompatible according to ISO 10993-5 standard, with cell viability above 70%.

### 3.7. Mathematical Analysis

This study investigates the mechanical properties of PLA-based polymer samples fabricated through the FDM method, focusing on flexural strength (σ_max_) and surface roughness (Ra), and relates them to manufacturing parameters using multivariate regression models. By assessing both the individual and interactive effects of manufacturing variables, namely infill ratio (X_1_, DO), layer thickness (X_2_, KK), and printing angle (X_3_, UA), the study explored how model behaviors varied across different material types.

The flexural strength (σ_max_) and surface roughness (Ra) performances σ_max_ of PLA, Re-PLA, St-PLA, and St-Re-PLA specimens were related to the production parameters using X_1_ multivariate regression models. When both individual and interactive contributions of production variables such as infill rate (X_1_, DO), layer thickness (X_2_, KK), and printing orientation (X_3_, UA) were evaluated, different model behaviors were investigated according to the material type. Multivariate second-order polynomial regression models were developed depending on the three basic production parameters affecting flexural strength and surface roughness. Taking these models into account, general flexural strength (σ_max_) and general surface roughness (Ra) models were also developed. The flexural strength (σ_max_) models are given in Table 4, and the surface roughness (Ra) models are given in Table 5, along with performance evaluation and analysis comments.

Table 4 shows that the performance evaluations of the flexural strength models of PLA, Re-PLA, St-PLA, and St-Re-PLA specimens were carried out using the coefficient of determination (R^2^), mean absolute error (MAE), and root mean square error (RMSE) criteria. The highest explanatory power (R^2^ = 0.87) was observed in the PLA model, whereas the St-Re-PLA model exhibited lower fit and higher variance (R^2^ = 0.46). The combined model obtained by averaging all PLA types provided a balanced generalization with a value of R^2^ = 0.64. The regression models obtained successfully demonstrated the effect of production parameters on mechanical behavior; layer thickness, in particular, stood out as the most significant influencing variable among all variants.

When Table 5 is examined, it is observed that the St-PLA group provided the highest explanatory power with R^2^ = 0.68, while the St-Re-PLA group had the lowest fit with R^2^ = 0.12. Layer thickness (X_2_) was the most decisive parameter for surface roughness in almost all variants, and the surface was observed to be wavier and irregular, especially in thicker layers.

The predictability of surface roughness decreased in recycled and sterilized PLA specimens. This is consistent with the literature that recycling weakens the polymer chain structure and thermal degradation causes microscopic defects on the surface.

In conclusion, although the modeling of Ra values was not as successful as that of mechanical strength, regression models yielded statistically reliable results, especially for certain groups such as the St-PLA variant. These findings demonstrate the influence of production parameters on surface quality and, in agreement with the literature, show that surface behavior can be quite variable [53].

When the mechanical and surface roughness modeling studies for PLA, Re-PLA, St-PLA and St-Re-PLA were evaluated, the flexural strength (σ_max_) and surface roughness (Ra) properties of PLA, Re-PLA, St-PLA, and St-Re-PLA specimens were investigated with multivariate second order regression models depending on the production parameters. While the explanatory power of the flexural strength models was observed (R^2^ ≥ 0.69), the surface roughness models had a lower explanatory power (R^2^ ≤ 0.34). This can be explained by the fact that while the mechanical properties show more direct and regular relationships with the production parameters, Ra is more susceptible to microstructural defects and external effects. In particular, layer thickness (X_2_) was the most decisive parameter for both strength and surface quality, while the printing orientation (X_3_) had an impact on bending strength but limited its effect on Ra. Recycling and sterilization processes increased variance, which in turn increased the margin of error, particularly in the Ra models. The results indicate that the mechanical strength of PLA-based parts produced with FDM can be predicted through parametric modeling, but surface roughness requires more complex modeling approaches.

In conclusion, because flexural strength in PLA specimens exhibits strong relationships with manufacturing parameters, regression modeling allowed for higher accuracy than surface roughness data. However, because surface roughness has more complex micro-level effects, more advanced, nonlinear approaches may be necessary.

The unstable response of recycled or sterilized polymer materials to the forming angle can be explained by orientation-dependent structural weaknesses. The deformation trends observed with increasing forming angle may be a result of internal structural irregularities in these materials [11,45]. Therefore, models developed should consider not only linear variables but also the thermomechanical history and anisotropic behavior of the material.

Surface roughness models have shown that classical regression techniques are inadequate with low R^2^ and high variance. In this context, spline regression, Random Forest (RF), Support Vector More flexible models such as Machines (SVM) are recommended [54].

The findings demonstrate the impact of FDM process parameters on both the mechanical and surface properties of polymer-based samples using a multivariate approach. The study makes a significant contribution to literature by providing a model-based comparative analysis of the responses of different material types to post-production processes (recycling, sterilization).

## 4. Conclusions

This conclusion synthesizes the experimental and analytical results spanning flexural strength, deformation, surface roughness, SEM morphology, degradation/cytotoxicity, and regression modeling to elucidate how infill rate, layer thickness, and printing orientation govern the performance of PLA, Re-PLA, St-PLA, and St-Re-PLA.

- It was observed that the infill rate, layer thickness, and printing orientation had significant effects on the flexural strength of PLA, Re-PLA, St-PLA, and St-Re-PLA specimens. As the infill rate increased in PLA and Re-PLA, the flexural strength values increased, while sterilization and recycling processes made the behavior of the materials more wavy. In St-PLA specimens, layer thickness was found to establish a critical threshold, while in St-Re-PLA, recycling and sterilization together led to negative outcomes. Overall, it was concluded that production parameters must be adjusted more carefully, particularly in recycled and sterilized biopolymers.
- In PLA specimens, the infill rate, layer thickness, and printing orientation had uniform and significant effects on deformation, while these effects were more limited in Re-PLA. In St-PLA and St-Re-PLA specimens, the results were more variable, with the effects of layer thickness and printing orientation becoming irregular. In general, sterilization and recycling processes make the deformation behavior of materials more unpredictable, while the most regular and high deformation was obtained in PLA specimens.
- Surface roughness tests showed that the surface became smoother in all specimen types as the infill rate increased. It was observed that the layer thickness first increased the surface roughness and then decreased it after a certain value. Printing orientation was the parameter with the most negative effect on all specimens, with surface marks becoming more pronounced at 45° and 90° angles. In general, the increase in roughness was more noticeable in PLA and St-PLA, while in St-Re-PLA, surface quality reached its best level at high infill rates.
- SEM images showed that as the infill rate increased, the voids in the specimens decreased and the adhesion between filaments improved. While a more brittle fracture behavior was observed in PLA and Re-PLA specimens, plastic deformation traces became more pronounced, and ductile fracture properties became more prominent in St-PLA and St-Re-PLA. These findings are consistent with the surface roughness results and reveal that sterilization and recycling processes significantly affect the fracture morphology of the material.
- In our tests, none of the PLA, Re-PLA, St-PLA, or St-Re-PLA specimens, whether filled at 25%, 50%, or 75%, showed any degradation at all. When we checked how well the materials worked with cells, PLA gave the best results, St-PLA was very close, St-Re-PLA was slightly lower, and Re-PLA showed the lowest values, but still remained non-cytotoxic.
- The regression analyses demonstrated that second-order polynomial models provide robust predictive capability for mechanical properties, particularly bending strength, with layer thickness emerging as the dominant process parameter for PLA. By contrast, surface roughness exhibited lower predictability and higher error margins, underscoring the limitations of classical regression approaches in capturing microstructural complexities. These findings affirm the adequacy of polynomial regression for mechanical performance prediction, while simultaneously indicating the necessity of more advanced modeling strategies for surface quality assessment.
- Considering that steam sterilization adversely affected the performance of recycled specimens, future studies may investigate alternative sterilization methods such as ethylene oxide, gamma irradiation, or plasma treatment, which could potentially preserve the mechanical integrity and surface quality of PLA-based biomaterials.

## Figures and Tables

**Table 1:** Comparative summary of studies on the effects of steam sterilization and manufacturing parameters on the mechanical properties of PLA and Re-PLA produced by 3D printing.

Ref. No	Material	Production Parameters	Sterilization Method	Characterization Studies
[19]	PLA, Re-PLA	Layer thickness and infill density.	-	Tensile, compression, bending, stress, strength and surface quality.
[20]	PLA	İnfill density, raster angle, and printing speed.	-	Optimization and ANOVA analysis.
[21]	PLA	Infill density.	-	Bending strength, tensile strength and mechanical properties.
[22]	Bronze/PLA Composite	Printing orientation.	-	Mechanical properties, tensile strength, tribological behavior (friction, wear depth, stick-slip), surface roughness, and hardness.
[23]	Re- (PLA, ABS, PET, HDPE)	Manufacturing methods (FDM, HME, FPF) and recycling.	-	Tensile strength, flexibility, UV sensitivity, and biodegradability.
[17]	PLA	Thermomechanical behavior.	Steam	Deterioration and deformation.
[18]	PLA, PET-G, PEEK	Different polymer type.	Steam	Strength differences depending on material type.
[10]	PLA+ Hidroksiapatit	Surface treatment.	Steam s	FTIR, Gel Permeation Chromatography, and Differential scanning calorimetry (DSC).
[24]	PLA	Infill density.	Steam	Tensile strength (infill effect).
[25]	PLA	Layer thickness and build size.	Steam	Geometric deformation (axial deviation analysis), contamination test (sterility assessment by color change).
In this study	PLA, Re-PLA, St-PLA, St-Re-PLA	Infill rate, layer thickness, printing orientation, and recycling.	Steam	Bending strength, deformation, surface roughness, SEM, degradation and cytotoxic.

**Table 2:** Materials and their sources used in the study.

Material	Purpose of Use	Origin
PLA-PLUS filament	3D printing (base material)	ELAS-3B (Kocaeli, Turkey)
Recycled PLA (Re-PLA)	Produced from defective PLA-PLUS parts	Extrusion via Rondol Microlab twin screw extruder.
NaOH (1%)	Cleaning of 3D printing waste	Kimyalab (Istanbul, Turkey)
Active surface solvent (3%)	Cleaning of 3D printing waste	Kimyalab (Istanbul, Turkey)
Eryiğit ERS 6613 D Autoclave	Sterilization process	Eryiğit (Istanbul, Turkey)
Phosphate-buffered saline (PBS)	Degradation tests	Sigma-Aldrich (Istanbul, Turkey)
L929 mouse fibroblast cells	Cytotoxicity tests	Merck (Istanbul, Turkey)
DMEM/F12 medium	Cell culture medium	Sigma-Aldrich (Istanbul, Turkey)
Dimethyl sulfoxide (DMSO)	Solubilization of formazan crystals	Merck

**Table 3:** Experimental results of maximum flexural strength (σmax), maximum deformation (δ_max_), and surface roughness (Ra) for PLA, Re-PLA, St-PLA, and St-Re-PLA specimens.

Production Parameters			Max. Flexural Strength, Mpa				Max. Deformation				Surface Roughness, µm			
**Infill Rate, %**	**Layer Thickness,** **mm**	Printing Orientation, °	PLA	Re-PLA	St-PLA	St-Re-PLA	PLA	Re-PLA	St-PLA	St-Re-PLA	PLA	Re-PLA	St-PLA	St-Re-PLA
25	0.15	0	53.4	65.0	65.4	68.4	10.1	9.6	6.1	8.6	4.1	6.5	3.7	7.4
		45	49.5	62.1	72.6	67.2	8.5	10.5	6.1	7.7	5.2	6.2	4.5	6.5
		90	65.4	76.2	57.0	64.8	24.0	18.2	4.8	6.6	4.9	8.0	7.6	7.0
	0.2	0	60.3	70.8	49.2	49.8	9.7	12.2	5.3	6.6	3.4	7.7	6.6	7.3
		45	60.9	70.5	57.6	63.0	9.6	12.9	5.1	7.8	6.5	5.4	3.7	11.5
		90	66.9	70.2	46.8	46.2	13.0	19.3	6.7	5.3	6.0	4.6	4.9	7.6
	0.25	0	65.7	72.6	46.2	57.6	9.8	13.4	7.5	6.9	4.0	3.5	5.4	7.8
		45	66.6	76.2	47.4	49.2	10.9	13.0	9.1	8.6	5.7	5.3	6.7	8.1
		90	65.1	66.6	42.6	45.6	13.3	12.6	6.2	9.1	5.8	8.6	5.9	5.4
50	0.15	0	57.6	63.9	71.4	70.8	12.1	11.7	6.9	8.3	3.2	7.7	4.4	3.7
		45	60.6	73.5	60.0	51.0	12.3	14.9	5.8	9.6	7.0	6.3	5.7	7.1
		90	65.7	69.3	58.2	61.2	24.4	18.4	5.5	8.7	4.8	4.2	6.7	7.3
	0.2	0	65.7	69.0	45.6	67.2	13.0	12.3	6.6	7.2	4.6	7.0	3.4	7.5
		45	56.7	72.0	39.0	69.0	9.2	13.9	9.3	7.1	5.1	5.7	6.7	8.6
		90	67.2	67.5	48.6	60.0	18.6	15.4	8.3	6.4	4.7	7.6	6.5	9.4
	0.25	0	70.5	87.9	51.6	55.2	12.8	14.3	9.1	12.0	5.3	6.6	6.3	6.7
		45	70.5	76.2	49.8	51.6	12.8	13.6	7.7	8.5	5.7	5.0	3.7	3.2
		90	67.8	78.9	39.0	65.4	24.7	14.4	9.3	7.2	2.9	7.1	4.9	7.6
75	0.15	0	75.0	80.1	75.6	53.4	13.1	13.2	5.9	8.8	4.8	3.3	5.6	5.8
		45	69.9	72.3	50.4	57.0	11.8	13.5	7.8	9.9	4.1	7.0	6.6	8.1
		90	75.3	75.9	61.8	66.6	16.1	17.7	5.4	6.4	3.6	4.9	3.7	6.4
	0.2	0	70.8	75.6	43.2	46.8	12.2	12.2	6.3	11.2	4.2	5.6	4.9	6.5
		45	70.5	76.8	46.2	56.4	11.2	12.2	6.7	8.3	4.1	6.3	5.4	3.0
		90	74.7	78.9	64.2	63.6	18.4	18.8	7.2	14.4	5.2	7.5	6.7	7.3
	0.25	0	73.8	90.0	60.6	58.2	12.1	17.0	6.9	9.9	5.5	6.7	3.9	5.5
		45	72.9	87.0	45.0	69.6	11.8	12.9	5.8	6.9	4.9	5.9	4.4	7.6
		90	75.6	69.0	60.0	59.4	22.2	30.1	5.8	7.1	5.9	6.3	6.7	6.8

**Table 4:** Flexural strength (σ_max_) modeling study of PLA, Re-PLA, St -PLA and St -Re-PLA specimens and performance evaluation of the modeling study.

**Model**	Equation	R^2^	MAE	RMSE	Analysis Comment
σ_max_ PLA	$\begin{array}{c} Y=0.2087\cdot X_{1}+68.8333\cdot X_{2}+0.0959\cdot \\ X_{3}+0.0042\cdot X_{1}^{2}-1.8000\cdot X_{1}\cdot X_{2}-\\ 0.0009\cdot X_{1}\cdot X_{3}+300.0000\cdot X_{2}^{2}-0.8111\cdot \\ X_{2}\cdot X_{3}+0.0017\cdot X_{3}^{2} \end{array}$	0.87	1.92	2.41	The model has high explanatory power. The effect of layer thickness is evident.
σ_max_ Re-PLA	$\begin{array}{c} Y=0.0088\cdot X_{1}-221.0000\cdot X_{2}+0.4433\cdot \\ X_{3}+0.0017\cdot X_{1}^{2}+0.3733\cdot X_{1}\cdot X_{2}-\\ 0.0020\cdot X_{1}\cdot X_{3}+891.1111\cdot X_{2}^{2}-1.7926\cdot \\ X_{2}\cdot X_{3}-0.0002\cdot X_{3}^{2} \end{array}$	0.69	3.30	3.81	For Re-PLA, the model is moderately explanatory; the X_2_^2^ effect is dominant.
σ_max_ St -PLA	$\begin{array}{c} Y=-1.0900\cdot X_{1}-1487.0000\cdot X_{2}-\\ 0.3793\cdot X_{3}+0.0058\cdot X_{1}^{2}+2.4400\cdot X_{1}\cdot \\ X_{2}+0.0016\cdot X_{1}\cdot X_{3}+2973.3333\cdot X_{2}^{2}+\\ 0.6889\cdot X_{2}\cdot X_{3}+0.0014\cdot X_{3}^{2} \end{array}$	0.61	5.32	6.27	For St-PLA the X_2_^2^ effect is very high but the overall performance of the model is poor.
σ_max_ St -Re-PLA	$\begin{array}{c} Y=-0.3680\cdot X_{1}-497.6667\cdot X_{2}-\\ 0.1563\cdot X_{3}+0.0053\cdot X_{1}^{2}+3.8800\cdot X_{1}\cdot \\ X_{2}+0.0037\cdot X_{1}\cdot X_{3}+626.6667\cdot X_{2}^{2}-\\ 0.0222\cdot X_{2}\cdot X_{3}+0.0002\cdot X_{3}^{2} \end{array}$	0.46	4.53	5.68	The St-Re-PLA model shows greater uncertainty and higher variance.
Unified PLA	$\begin{array}{c} Y=-0.3101\cdot X_{1}-534.2083\cdot X_{2}+\\ 0.0009\cdot X_{3}+0.0016\cdot X_{1}^{2}+1.2233\cdot X_{1}\cdot \\ X_{2}+0.0006\cdot X_{1}\cdot X_{3}+1197.7778\cdot X_{2}^{2}-\\ 0.4843\cdot X_{2}\cdot X_{3}+0.0007\cdot X_{3}^{2} \end{array}$	0.64	1.93	2.51	The generalized model is balanced but has lower explanatory power than the individual PLA models.

**Table 5:** Surface roughness (Ra) modeling study of PLA, Re-PLA, St-PLA and St-Re-PLA specimens and performance evaluation of the modeling study.

**Model**	Equation	R^2^	MAE	RMSE	Analysis Comment
Ra PLA	$\begin{array}{c} Y=-0.0388\cdot X_{1}-1.5439\cdot X_{2}+0.0677\cdot X_{3}+\\ 0.0001\cdot X_{1}^{2}+0.1785\cdot X_{1}\cdot X_{2}-0.0004\cdot X_{1}\cdot \\ X_{3}+1.2667\cdot X_{2}^{2}-0.0517\cdot X_{2}\cdot X_{3}-0.0004\cdot \\ X_{3}^{2} \end{array}$	0.34	0.62	0.80	The model has very limited explanatory power. Layer thickness has a negative effect.
Ra Re-PLA	$\begin{array}{c} Y=-0.0529\cdot X_{1}+20.3478\cdot X_{2}-0.0526\cdot \\ X_{3}+0.0004\cdot X_{1}^{2}+0.4667\cdot X_{1}\cdot X_{2}-0.0000\cdot \\ X_{1}\cdot X_{3}+129.4222\cdot X_{2}^{2}+0.2047\cdot X_{2}\cdot X_{3}+\\ 0.0002\cdot X_{3}^{2} \end{array}$	0.21	0.95	1.17	Low explanatory power; Re- PLA has high variance, X_2_ effect is dominant. Variance increased substantially due to recycling. Model is weak.
Ra St-PLA	$\begin{array}{c} Y=0.0392\cdot X_{1}+23.0367\cdot X_{2}+0.0226\cdot X_{3}+\\ 0.0000\cdot X_{1}^{2}-0.2042\cdot X_{1}\cdot X_{2}-0.0000\cdot X_{1}\cdot \\ X_{3}+23.3111\cdot X_{2}^{2}-0.0931\cdot X_{2}\cdot X_{3}+0.0001\cdot \\ X_{3}^{2} \end{array}$	0.16	0.93	1.12	The effect of X_2_ is in the opposite direction.
Ra St-Re-PLA	$\begin{array}{c} Y=-0.0595\cdot X_{1}+180.0972\cdot X_{2}+0.0301\cdot \\ X_{3}+0.0003\cdot X_{1}^{2}-0.0587\cdot X_{1}\cdot X_{2}-0.0004\cdot \\ X_{1}\cdot X_{3}+428.8444\cdot X_{2}^{2}-0.1442\cdot X_{2}\cdot X_{3}-\\ 0.0001\cdot X_{3}^{2} \end{array}$	0.25	1.11	1.50	Highest variance. (highest deviation here) The combined effect reduces model performance.
Unified PLA	$\begin{array}{c} Y=-0.0280\cdot X_{1}+55.4844\cdot X_{2}+0.0170\cdot X_{3}+\\ 0.0000\cdot X_{1}^{2}+0.0956\cdot X_{1}\cdot X_{2}-0.0000\cdot X_{1}\cdot \\ X_{3}+145.7111\cdot X_{2}^{2}-0.0211\cdot X_{2}\cdot X_{3}-\\ 0.0001\cdot X_{3}^{2} \end{array}$	0.29	0.51	0.62	The generalized PLA model can predict average behavior more stably across variants (lower MAE), but is weaker than the PLA model in explaining the internal variance of PLA (lower R^2^).

## Data Availability

The original contributions presented in this study are included in the article. Further inquiries can be directed to the corresponding author(s).
